# The epidemiology of fatal cyclist crashes over a 14-year period in Alberta, Canada

**DOI:** 10.1186/s12889-015-2476-9

**Published:** 2015-11-17

**Authors:** Lindsay Gaudet, Nicole T. R. Romanow, Alberto Nettel-Aguirre, Donald Voaklander, Brent E. Hagel, Brian H. Rowe

**Affiliations:** School of Public Health, University of Alberta, Edmonton, Canada; Department of Emergency Medicine, University of Alberta, 1G1.43 WMC, 8440-112 Street NW, Edmonton, AB T6G 2B7 Canada; Faculty of Kinesiology, University of Calgary, Calgary, AB Canada; Department of Pediatrics, Cumming School of Medicine, University of Calgary, Calgary, AB Canada; Department of Community Health Sciences, Cumming School of Medicine, University of Calgary, Calgary, AB Canada; Alberta Children’s Hospital Research Institute, University of Calgary, Calgary, AB Canada

**Keywords:** Bicycle, Fatality, Alberta

## Abstract

**Background:**

Cycling is a popular recreational activity and a common transportation option; however, cycling-related injuries can be fatal. There are few studies of cycling fatalities in Canada and none in a region as sparsely populated as Alberta.

**Methods:**

A chart review was conducted of cyclists involved in fatal crashes. Charts for deaths that occurred between 1998 and 2011 (inclusive) were identified and abstracted onto standardized forms. Personal characteristics and crash circumstances, including motor vehicle involvement, were collected; mechanisms of fatally injured cyclists across age groups were compared. Census data were used to calculate region-specific and provincial age-specific cycling fatality rates.

**Results:**

Charts from 101 deaths over 14 years were reviewed. Events mainly occurred during the summer. There were more fatalities in urban (64 [63 %]) than in rural settings. Collisions with motor vehicles and cyclist-only crashes accounted for 68 and 15 % of cycling fatalities, respectively. Most (87 %) deceased cyclists were male, and the median age was 47 years (inter-quartile range: 25, 58). The population-based fatality rate over the study period was highest among deceased cyclists older than 65. Helmet use was reported in 26 (26 %) cases and increased with age. Alcohol use was detected in 25 (25 %) cases.

**Conclusions:**

Fatal cycling crashes in Alberta typically involve adults riding on urban roads and collisions with motor vehicles. While helmet legislation has reduced non-fatal cycling head injuries, deaths may be further prevented by physical separation of cyclists and motor vehicles and avoidance of substance use while operating bicycles.

**Electronic supplementary material:**

The online version of this article (doi:10.1186/s12889-015-2476-9) contains supplementary material, which is available to authorized users.

## Background

Cycling is a popular recreational activity that has both health and environmental benefits [1]. Despite the benefits, cycling can be dangerous, and cycling crashes can be fatal. While cycling fatalities are rare events, cyclists are killed at higher rates than other road users [2]. Cycling fatalities also have higher per-event medical costs than nonfatal injuries [3].

Previous studies of bicycle fatalities conducted in the United States have found that fatally injured cyclists are usually male, often adult, and rarely wearing a helmet when they crash [4–6]. The relationships of cyclist and environmental factors to the mechanisms of cycling-related deaths among Canadian cyclists have not been thoroughly investigated; however, several recent studies evaluated helmet effectiveness for prevention of cycling-related fatalities and found decreased occurrence of head injury among severely and fatally injured cyclists [7–10]. In addition, the prevalence of cycling fatality risk factors may vary with age, [11] but these differences remain relatively unstudied. Many governments are making efforts to increase cycling among residents; however, the primary barrier is the perception that cycling is dangerous [12]. Understanding the factors that contribute to fatal cycling crashes and their difference between age groups will assist decision-makers to develop tailored interventions for prevention of cycling fatalities, a decrease in which may mitigate the perceived danger of cycling and thereby encourage more people to cycle.

Canada is a large, geographically diverse country with both dense urban and sparse rural areas, and a disperate climates ranging from warm oceanic to sub-arctic. Most studies of Canadian cycling fatalities have used data from regions that are more densely populated and with warmer, wetter climates than Alberta, namely the province of Ontario and urban centers in southern British Columbia [7, 9, 13–16]. This is the first study to examine the mechanisms and epidemiological factors (e.g., environmental and demographic) contributing to cycling fatalities in Alberta, a sparsely populated, mostly rural region with a cold northern climate.

## Methods

### Study design

This is a retrospective chart review. Ethical approval to conduct the study was received from the University of Alberta Health Research Ethics Board.

### Data collection

Case files from Alberta’s Office of the Chief Medical Examiner (OCME) were used. Approximately 20 % of all deaths in Alberta are investigated by the OCME, including all deaths that are the result of an accident [17]. Case files for the years 1998 to 2011, inclusive, coded with any of “MV bicycle”, “fall”, “sporting”, or “other” were manually reviewed by OCME staff to identify bicycle-related deaths. Relevant cases were abstracted by two trained research staff onto standardized forms. Information about demographics, environmental (e.g., location, date and time, lighting) and cyclist (e.g., clothing, helmet use, alcohol or drug use) factors, as well as injury data, were collected and entered into a purpose-built Microsoft Excel database.

### Data sources

All available records within OCME case files were used to complete the form. Event circumstances, including action of involved parties and details on the deceased cyclist, the bicycle, and the scene of the crash, were determined from incident summaries within emergency department (ED) charts, paramedic reports and local police or Royal Canadian Mounted Police (RCMP) reports, including scene photographs (*n* = 6). Previous studies have reported that helmet use by fatally injured cyclists is rare, [4, 7, 16] and helmet use in Alberta during the study period was generally low; [18] therefore, deceased cyclists were assumed not to have been wearing a helmet unless specifically stated. Injury details were collected from the medical examiner (ME) reports, nursing notes, medical consultant reports, ED charts, and paramedic reports. Evidence of alcohol and prescription and illicit drug use was collected from OCME and police toxicology reports or witness statements.

In Alberta, all traffic collisions involving a death must be reported to police. Therefore, information about actions of motorist(s) involved in a fatal motor vehicle-cyclist collision (MVCC) was obtained from RCMP or local police reports. MVCCs were categorized according to the mechanisms defined by others: mid-block ride-out, cyclist inattention, motorist inattention, other, or unknown/unclear [16].

### Case mapping and event rate calculations

Fatal crashes were mapped into one of six regions (Edmonton region, Calgary region, Northern Alberta, Central Alberta, Southern Alberta, and Alberta Rockies) based on location of the crash using the open-source OpenJUMP geographical information software package (Vivid Solutions, Victoria, BC) and 2006 census sub-division digital boundary data (Additional file 1: Figure S1) [19]. Regional and age-specific cycling fatality rates per million population were calculated by dividing the number of events per region by person-years during the study period, estimated by multiplying the population of each region from the 2006 Statistics Canada census data (the census data closest to the mid-point of the study) by the study duration (14 years).

### Statistical analysis

The numbers of fatally injured cyclists with injuries to each anatomical area (head, neck/spine, trunk, extremities) are reported; some deceased cyclists sustained injury to more than one area of the body. MVCC events were compared to all non-MVCC events on cyclist demographics (% male, age), location (urban vs. rural, road vs. non-road), reported helmet use, alcohol/drug use, light condition, weather conditions, and injuries. An event was classified as “urban” if it occurred within the legal limits of a city or town; all other settings were considered rural. Urban events were compared to rural events on cyclist demographics (% male, age), reported helmet use, alcohol/drug use, light condition, weather conditions, and injuries. Deceased cyclists were grouped into five age categories: <10 years, 10–19 years, 20–44 years, 45–64 years, and ≥65 years. Age groups were compared on reported helmet, alcohol, and illicit drug use, and injuries.

Statistical analyses were performed using STATA Statistical Software Release 12.0 (Stata Corporation; College Station, TX, USA). Numerical data are reported as medians and interquartile ranges (IQR). Proportions were calculated for categorical variables. Differences in sub-groups were tested using Fisher’s exact test; *p* ≤ 0.05 was considered statistically significant.

## Results

The OCME identified 106 cycling-related fatalities that occurred between February 1998 and October 2011. Five files were excluded because they did not describe the event, leaving 101 cases for analysis.

Deceased cyclists were mostly male (87 %) and mainly adult (81 %) (Table 1). Cyclists most often died at the scene (40 %) or after hospital admission (40 %) (Table 1). Helmet use was reported in 25 (25 %) and alcohol use in 26 (26 %) cases (Table 1).

**Table 1:** Demographic details of 101 fatally injured cyclists in Alberta, Canada

Characteristic	n (%)
	*N* = 101
Male	88 (87)
Age (years)	
< 10	7 (7)
10–19	12 (12)
20–44	31 (31)
45–64	31 (31)
≥ 65	20 (20)
Helmet use	26 (26)
Alcohol use	25 (25)
BAC^a^ ≥ 0.08 %	18 (18)
Unknown	3 (3)
Unknown	10 (10)
Illicit drug use	14 (14)
Unknown	33 (33)
Prescription drug use	7 (7)
Unknown	35 (35)
Time of death	
Dead at scene	41 (41)
Transport but DOA^b^	7 (7)
Transport but died in ED^c^	13 (13)
Died after admission to hospital	40 (40)

Percentages may not sum to 100 due to rounding

^a^ *BAC* blood alcohol concentration

^b^ *DOA* dead on arrival

^c^ *ED* emergency department

The number of fatal cycling crashes varied between years from a low of three events in 1998 to a high of 12 events in 2007; no trend in events per year was detected by a non-parametric test for trend (*p* = 0.4). Fatal crashes mainly occurred between May and September, and peaked in September (21 [21 %]). Most crashes occurred during the day, and crashes mainly occurred on a weekday (Table 2). Nine (9 %) events occurred during peak traffic (6:30 AM to 8:30 AM and 4 PM to 6 PM, Monday to Friday). The majority of crashes occurred on a road, and poor lighting and inclement weather were infrequent (Table 2). The majority of fatal crashes were MVCCs (Table 2); “other” mechanisms included a collision with a train and three collisions with urban rail transit. Of the 67 MVCC-related deaths, eight (12 %) were midblock ride-outs, 23 (34 %) were due to cyclist inattention, 25 (37 %) were due to motorist inattention, and 11 (16 %) were categorized as other/unknown. MVCCs occurred more frequently than non-MVCC crashes on roads (*p* < 0.001; Table 3) and more often in poor lighting (*p* = 0.029; Table 3). Incidence of trunk injury was higher among cyclists killed in MVCCs than non-MVCC crashes (Table 3). Reported helmet use did not differ with crash mechanism; however, evident cyclist alcohol use was present more often in MVCCs than in non-MVCC crashes (*p* < 0.001; Table 3).

**Table 2:** Descriptive characteristics of 101 fatal bicycle crashes in Alberta, Canada

Characteristic	n (%)
	*N* = 101
Time of crash	
0601–1200	20 (20)
1201–1800	38 (38)
1801–0000	28 (28)
0001–0600	9 (9)
Unknown	6 (6)
Day of the week	
Monday	13 (13)
Tuesday	14 (14)
Wednesday	11 (11)
Thursday	14 (14)
Friday	16 (16)
Saturday	19 (19)
Sunday	14 (14)
Location	
Urban	64 (63)
Rural	31 (31)
Undetermined	6 (6)
Route type	
Road	77 (76)
Sidewalk/Pathway	6 (6)
Trail/off-road	10 (10)
Other	4 (4)
Unknown	4 (4)
Surface type	
Pavement	80 (80)
Gravel	7 (7)
Dirt/grass	4 (4)
Unable to determine	10 (10)
Mechanism	
MVCC^a^	67 (66)
Fell or thrown from bicycle	15 (15)
Collision with stationary object	7 (7)
Other	12 (12)

Percentages may not sum to 100 due to rounding

^a^ *MVCC* motor vehicle-cyclist collision

**Table 3:** Cyclists and crash characteristics by mechanism for 101 fatal bicycle crashes in Alberta, Canada

Characteristic	Non-MVCC^a^ (*N* = 34)	MVCC *N* = 67)	Fischer’s Exact *p*-value
Location	n (%)	n (%)	
Urban	27 (79)	37 (55)	0.030
Rural	5 (15)	26 (39)	
Undetermined	2 (6)	4 (6)	
Environment			
On road	14 (41)	63 (94)	<0.001
Poor lighting	6 (18)	20 (30)	0.029
Cyclist			
Helmet use	8 (24)	18 (27)	0.812
Alcohol use	13 (38)	12 (18)	0.031
BAC^b^ > 0.08 g/dL (17 mmol/L)	8 (24)	10 (15)	0.378
Drug use	4 (12)	10 (15)	0.448
Type of injury			
Head injury	28 (82)	48 (72)	0.330
Trunk injury	10 (29)	47 (70)	<0.001
Spine injury	11 (32)	15 (22)	0.337
Limb injury	17 (50)	51 (76)	0.013

Percentages may not sum to 100 due to rounding

^a^ *MVCC* motor vehicle-cyclist collision

^b^ *BAC* blood alcohol content

Table 4 presents the number of events and rate per million population in each region. Rural regions generally had higher rates of events compared with urban regions (Table 4); the event rate was highest in the Alberta Rockies. Table 4 also presents the population-based rates of events by age-group, which increased with age.

**Table 4:** Regional and age-based rates of fatal bicycle injuries. Rates were calculated by dividing the number of events per region by person-years during the study period, which were estimated by multiplying the population of each region by the study duration (14 years)

	No. events (%)	Population^a^	Events per 1,000,000 inhabitants per year
Region			
Edmonton	30 (30)	1,034,945	2.1
Calgary	26 (26)	1,079,310	1.7
Northern Alberta	13 (13)	328,073	2.8
Central Alberta	11 (11)	240,368	3.3
Southern Alberta	13 (13)	272,017	3.4
Alberta Rockies	8 (8)	35,983	15.9
Age category			
< 10	7 (7)	406,705	1.2
10 to 19	12 (12)	462,705	1.9
20 to 44	31 (31)	1,232,350	1.8
45 to 64	31 (31)	835,170	2.7
≥ 65	20 (20)	353,410	4.0

Percentages may not sum to 100 due to rounding

^a^Population data taken from the Statistics Canada 2006 Census

While the majority of crashes occurred in an urban location (Table 5), the proportion of fatalities due to MVCCs was higher in rural areas than in urban areas (84 % vs. 58 %; *p* = 0.03). Alcohol use was more frequent among cyclists killed in urban compared with rural crashes (Table 5; *p* = 0.048). While the frequency of reported helmet use was similar (Table 5; *p* = 0.22), head injuries occurred more frequently among cyclists killed in urban crashes compared with rural crashes (Table 5; *p* < 0.001)

**Table 5:** Cyclists and crash characteristics by location of the crash event for 101 fatal bicycle crashes in Alberta, Canada

Characteristic	Urban (*N* = 64)	Rural (*N* = 31)	Undetermined (*N* = 6)	Fischer’s Exact *p*-value
Environment	n (%)	n (%)	n (%)	
On road	46 (72)	26 (84)	5 (83)	0.500
Poor lighting	21 (33)	5 (16)	2 (33)	0.322
Cyclist				
Helmet use	14 (22)	9 (29)	3 (50)	0.222
Alcohol use	20 (31)	3 (10)	2 (33)	0.048
BAC^b^ > 0.08 g/L	14 (22)	2 (6)	2 (33)	1.00
Drug use				0.171
Age				
< 10	4 (6)	3 (10)	0	0.980
10–19	7 (11)	4 (13)	1 (17)	
20–44	22 (34)	8 (26)	1 (17)	
45–64	18 (28)	10 (32)	3 (50)	
≥ 65	13 (20)	6 (20)	1 (17)	
Type of injury				
Head injury	56 (88)	15 (48)	5 (83)	<0.001
Trunk injury	31 (48)	23 (74)	3 (50)	0.053
Spine injury	14 (22)	11 (35)	1 (17)	0.362
Limb injury	45 (70)	20 (65)	3 (50)	0.491

Percentages may not sum to 100 due to rounding

^a^ *BAC* blood alcohol content

Reported helmet use increased with age; deceased children and adolescents were rarely reported to be wearing a helmet and elderly deceased cyclists were most often reported to be wearing a helmet (Table 6; *p* = 0.077). Detected alcohol use was highest among deceased adolescents (Table 6). Most (18/25) deceased cyclists with indication of alcohol were intoxicated (BAC > 0.08 g/dL); the median BAC was 0.17 g/dL (IQR: 0.13, 0.23). All deceased cyclists 20–44 years old with evident alcohol use (*n* = 9) were intoxicated (Table 6). Incidence of MVCC compared to non-MVCC crashes was higher among deceased cyclists <20 years old than ≥20 years old, but was not significant (*p* = 0.101). Motorist inattention was involved in the deaths of most children and a notable fraction of adults 20–64 years old (Table 6). All cyclists who were killed after collision with a stationary object were ≥20 years old (Table 6).

**Table 6:** Cyclist characteristics and crash characteristics for 101 fatal bicycle crashes in Alberta, Canada by age

	<10	10–19	20–44	45–64	≥65	Fisher’s Exact *p*-value
	(*N* = 7)	(*N* = 12)	(*N* = 31)	(*N* = 31)	(*N* = 20)	
	n (%)	n (%)	n (%)	n (%)	n (%)	
Male	4 (57)	12 (100)	27 (87)	27 (87)	18 (90)	0.161
Alcohol use	0	5 (42)	9 (29)	10 (32)	1 (5)	0.035
BAC^a^ >0.08 %	0	4 (33)	9 (100)	5 (50)	0	0.029
Illicit drug use	0	3 (25)	9 (29)	2 (6)	0	0.0984
Helmet	1 (14)	1 (8)	8 (26)	6 (19)	10 (50)	0.077
Location						
Urban	4 (57)	7 (58)	22 (71)	18 (58)	13 (65)	0.955
Rural	3 (43)	4 (33)	8 (26)	10 (32)	6 (30)	
Unknown	0 (0)	1 (8)	1 (3)	3 (10)	1 (5)	
Environment						
On road	6 (86)	11 (92)	21 (68)	23 (74)	16 (80)	0.575
Darkness	1 (14)	5 (42)	12 (39)	9 (29)	1(5)	0.044
Mechanism						
Cyclist fall	0	0	6 (19)	6 (19)	3 (15)	0.390
MVCC^b^	7 (100)	9 (75)	20 (65)	19 (61)	12 (60)	
Midblock ride-out	1 (14)	2 (17)	3 (15)	1 (5)	1 (5)	0.545
Cyclist inattention	2 (29)	4 (44)	4 (20)	6 (32)	7 (35)	
Motorist inattention	4 (57)	1 (8)	9 (45)	7 (37)	4 (20)	
Unclear/Unknown	0	2 (17)	4 (20)	5 (26)	0	
Struck object	0	0	4 (13)	2 (6)	1 (5)	
Other	0	3 (25)	1 (3)	4 (13)	4 (20)	
Injury						
Head injury	5 (71)	10 (83)	24 (77)	23 (74)	14 (70)	0.929
Trunk injury	4 (57)	9 (75)	18 (58)	15 (48)	11 (55)	0.655
Spine injury	1 (14)	1 (8)	11 (35)	8 (26)	5 (25)	0.475
Limb injury	5 (71)	9 (75)	22 (71)	20 (65)	12 (60)	0.895

Percentages may not sum to 100 % due to rounding

^a^ *BAC* blood alcohol concentration

^b^ *MVCC* motor vehicle-cyclist collision

Fatally-injured cyclists who had evidence of alcohol consumption were less frequently reported to be wearing a helmet than deceased cyclists without documented alcohol use (4 % vs. 33 %; *p* = 0.003). More deceased cyclists who crashed in the dark had consumed alcohol compared with those who crashed in daylight (15/25 vs 8/67; *p* < 0.001).

## Discussion

This is the first report of cycling fatalities in Alberta, a province with two large urban areas, five mid-sized regional centers, and a large number of small towns across sparsely populated rural areas. Fatally injured cyclists were most often adult males and commonly sustained their injuries following a collision with a motor vehicle. Reported helmet use was infrequent, and 76 % of fatally injured cyclists suffered a head or brain injury. Fatal cycling collisions most often occurred in urban locations, on roads, and during times of good light and fair weather (i.e., when cycling is most frequent).

Notable differences were observed in deceased cyclists and crash circumstances between urban and rural areas. Although most fatal cycling crashes in Alberta occurred in urban areas, the rates of cycling deaths per 1,000,000 inhabitants per year were noticeably higher in rural regions where cyclists may ride more often on highways due to lack of other available routes. Additionally, health resources are typically more limited in rural regions which may result in a longer delay between crash occurrence and receiving advanced trauma care than in urban areas [20]. The fatality rate was especially high in the Alberta Rockies, which has a very small population and is popular with cycle-tourists. Future research should examine the burden on local health systems due to injuries occurring in non-residents, as this could have implications for where to target injury prevention strategies. Cyclists killed in urban areas more frequently had evidence of alcohol use than cyclists killed in rural areas. Cycling may be viewed by urban cyclists as a safe transportation alternative to driving after planned drinking; on the other hand, cycling after drinking is not as feasible in rural areas due to the often long travel distances.

Canadian cycling fatalities have been previously examined in British Columbia and Ontario. In British Columbia, cyclists were at higher risk of being struck by a motor vehicle and fatal injury than in-line skaters and skateboarders [13]. In Ontario, young (<10 years old) and older (≥45 years old) cyclists who died after a cycling crash more frequently made cycling errors that contributed to the crash than adolescents and younger adults, while 19–44 year olds were more frequently killed while cycling at night [16]. The most recent Ontario inquiry, which had similar findings to the current study, resulted in a Coroner’s Report and recommendations for safer cycling [7, 21].

Despite the provincial differences, these results from Alberta mainly agree with previous studies of Canadian cycling fatalities in that the majority of cyclist deaths involved males and were the result of collisions with motor vehicles [7, 16]. However, while previous reports from Ontario and the United States indicated that one-third of cycling fatalities occurred in children, [16, 22] only 19 % of deceased cyclists in the current report were under the age of 20. In our study, all children and most of adolescents died after a collision with a motor vehicle, which is consistent with other reports of pediatric cycling fatalities [15, 16]. It is possible that recently implemented interventions aimed to reduce distracted driving may also help to reduce cycling fatalities.

Cycling fatalities among children and adolescents have been decreasing nationally, which may result from improvements in the built environment and/or mandatory helmet legislation (MHL), which was introduced first in Canada in Ontario in 1998, in 2002 in Alberta (for cyclists <18 years only), and has since been introduced in other provinces. Although reported helmet use was low in this study, a larger proportion of fatally injured cyclists were wearing a helmet compared with older reports [4, 15, 16]. This increase is likely due to the influence of MHL, which (although targeted at under-age cyclists) has been shown to increase helmet use among all ages [18].

It is interesting to note that the population-based rate of fatal cycling events increased with age, but this observation must be interpreted with caution. Older cyclists may be less coordinated and frailer than younger cyclists and therefore more likely to be involved in a cycling crash and sustain more serious injuries; [23] however, data on cycling exposure including distance traveled, number of trips, and number of cyclists, are not readily available for Alberta, and it is possible (although unlikely) that older cyclists have a higher level of exposure to cycling which may bias the population-based rate upwards.

Alcohol use prior to fatal cycling events was particularly concerning. The correlation of alcohol consumption with non-use of protective devices such as helmets and seatbelts has been previously documented and is also indicated by our data, as deceased cyclists with evident alcohol use were less frequently reported to have been wearing a helmet than deceased cyclists without evident alcohol use [24]. The legal BAC limit for motorists in Alberta during the study period was 0.08 g/dL (17 mmol/L). Twenty-three of the 26 deceased cyclists with evident alcohol consumption, and all of those between 20 and 44 years old, were over the legal limit. Similar to a previous report, [25] in this study a larger proportion of deceased cyclists involved in non-MVCC collisions were intoxicated. Compared with the widespread knowledge of the effects and penalties of consuming alcohol before driving a motor vehicle, [26] cyclists may not recognize the danger of impairment while cycling and may use cycling as an alternative mode of transportation after social drinking, which is suggested by the high proportion of younger adult (20–44 years old) cyclists with evident alcohol use who were over the legal limit for operation of a motor vehicle. Additionally, drivers who lose their license due to impaired driving convictions are likely to turn to cycling as an alternative mode of transportation [27]. Drinking and biking has been identified as a potential issue elsewhere; [28] further study on the attitudes around drinking and cycling is needed to develop successful strategies to reduce injuries resulting from drinking and biking.

Cycling fatalities account for a disproportionate 3.2 % of Canadian road fatalities, though cyclists only make up 1.2 % of regular road users [12, 29]. Reports of cyclist crashes contribute to a lack of safety perceived by both cyclists and non-cyclists [12]. While the results presented here represent only one province, there are few other studies of cycling fatalities in regions with similar demographic and climactic characteristics, and the results presented here may be of interest to policy- and decision-makers in other regions with a low population densities and cold climates in Canada, such as Saskatchewan, Manitoba, and the Territories, and around the world (e.g., Northern mid-west US states, eastern Russia, etc.)

Future efforts to reduce cycling fatalities will require attention to interactions between motorists and cyclists. Prevention strategies that target risk-taking behaviors among cyclists, including education for cyclists on the potential dangers of drinking and cycling and encouraging all cyclists to wear a helmet should be explored. In addition, educational initiatives to instruct safe interactions between all types of road users should be implemented in Alberta, and other similar regions, to inform all road users on how to safely interact with each other [30]. These safety initiatives could help to reduce the public’s perception of cycling as a dangerous activity, and therefore help to decrease injuries, increase physical activity, and promote the uptake of active transportation.

### Limitations

The results of this exploratory study should be interpreted with caution, due to the lack of a control group. The retrospective methods employed in this study present several limitations. Relevant cases may have been missed due to miscoding, and OCME files are not available for cases in which an investigation, litigation or criminal proceedings are ongoing; however, given the small number of fatalities that occurred in the late fall and winter in other years, it is unlikely that a significant number of cases from 2011 were missed. The level of detail and missing data in the OCME files varied; thus the proportions listed here should be interpreted conservatively, as they may be underestimates. The rates calculated for the whole study period may over-estimate the annual cycling fatality rates prior to 2006 and under-estimate the annual rates after 2006 due to variation in the annual number of events and the change in Alberta’s population over the study period. Finally, the use of population as the denominator for the fatality rates here does not reflect the risk of death due to exposure to cycling.

## Conclusions

Cycling fatalities in Alberta are rare events that usually involve adults and are largely the result of a motor vehicle-cyclist encounter. Strategies to prevent cycling-related fatalities should include the physical separation of and promotion of safe interactions between motor vehicles and cyclists, interventions to increase helmet use by all ages, and education about the potentially devastating effect of alcohol use on the operation of a bicycle. Reducing fatalities will increase the perceived safety of cycling and should help increase cycling activity in both frequency of cycling and distance travelled.

## Additional file

Additional file 1: Figure S1. Population-based rates per million population per year of fatal bicycle crashes in Alberta from 1998 to 2011 by region. Numbers of events are in parentheses. Rates were calculated by dividing the number of events per region by person-years during the study period, which were estimated by multiplying the population of each region by the study duration (14 years). (PDF 199 kb)

## Abbreviations

BACDOA: Blood Alcohol concentrationDead on arrival
DOA: Dead on arrival
ED: Emergency department
ME: Medical examiner
MHL: Mandatory helmet legislation
MVCC: Motor vehicle/cyclist collision
OCME: Office of the chief medical examiner
RCMP: Royal Canadian mounted police
