# Supplementary material for: The epidemiology of fatal cyclist crashes over a 14-year period in Alberta, Canada
Source: BMC Public Health. 2015 Nov 17;15:1142. doi: 10.1186/s12889-015-2476-9 (PMC4650295; doi:10.1186/s12889-015-2476-9)
Supplement: Additional file 1: Figure S1. — Population-based rates per million population per year of fatal bicycle crashes in Alberta from 1998 to 2011 by region. Numbers of events are in parentheses. Rates were calculated by dividing the number of events per region by person-years during the study period, which were estimated by multiplying the population of each region by the study duration (14 years). (PDF 199 kb) [file 12889_2015_2476_MOESM1_ESM.pdf]

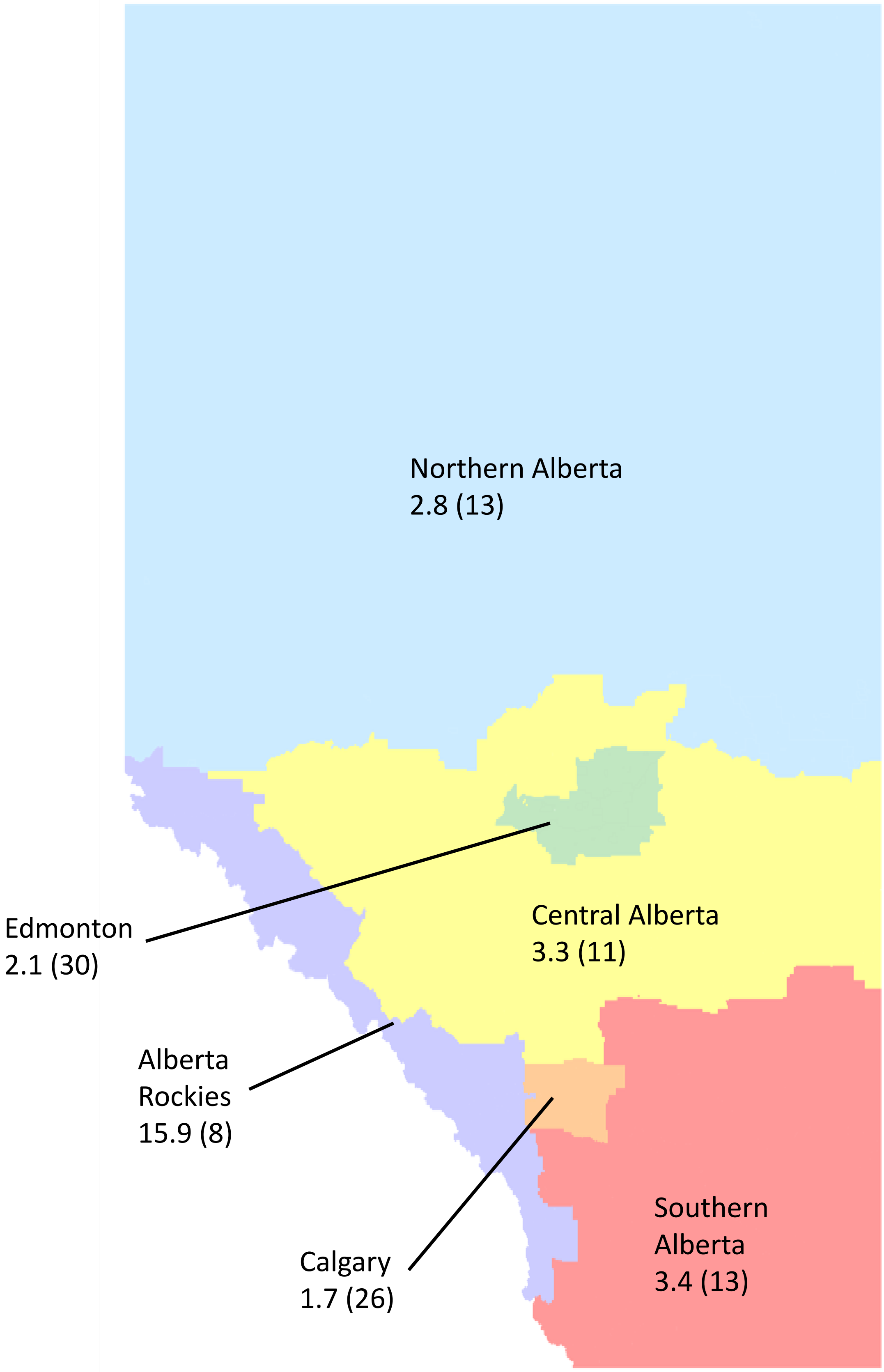

Supplementary Figure 1. Population-based rates per 1,000,000 population per year of fatal cycling crashes in Alberta from 1998 to 2011 by region. Numbers of events are in parentheses. Rates were calculated by dividing the number of events per region by person-years during the study period, which were estimated by multiplying the population of each region by the study duration (14 years)
